# Mechanobiological and Molecular Alterations in the Aging Dentin–Pulp Complex

**DOI:** 10.3390/life16050844

**Published:** 2026-05-20

**Authors:** Neshka Manchorova-Veleva, Mina Pencheva, David Baruh, Veselina Todorova, Lyubomir Vangelov, Evgeni Ivanov, Margarita Guenova

**Affiliations:** 1 Department of Operative Dentistry and Endodontics, Faculty of Dental Medicine, Medical University of Plovdiv, 4002 Plovdiv, Bulgaria; veselina.todorova@mu-plovdiv.bg (V.T.); lyubomir.vangelov@mu-polvdiv.bg (L.V.); 2 Department of Medical Physics and Biophysics, Faculty of Pharmacy, Medical University of Plovdiv, 4002 Plovdiv, Bulgaria; 3 Department of Software Engineering, Faculty of Mathematics and Informatics, Sofia University “St. Kliment Ohridski”, 1164 Sofia, Bulgaria; dbaruh@uni-sofia.bg; 4 Open Laboratory on Experimental Micro and Nano Mechanics (OLEM), Institute of Mechanics, Bulgarian Academy of Sciences, 1113 Sofia, Bulgaria; ivanov_evgeni@imbm.bas.bg; 5 Laboratory of Haematopathology and Immunology, National Specialized Hospital for Haematological Diseases, 1756 Sofia, Bulgaria; margenova@gmail.com

**Keywords:** dental pulp, aging, apoptosis, autophagy, biomarkers, nanoindentation, atomic force microscopystat

## Abstract

The dental pulp–dentin complex is a dynamic tissue system whose structure and biological functions evolve over time under physiological, molecular, and environmental influences. This study aimed to characterize age-related alterations in apoptotic, inflammatory, and autophagy-associated signaling pathways, alongside nanoscale mechanical changes, and to evaluate their potential impact on pulp tissue homeostasis and cellular adaptive capacity. Materials and Methods: Human teeth (n = 90) were divided into three age groups: young (≤17 years), mature (18–50 years), and old (>51 years). Immunohistochemistry was performed to assess the expression of CD34, BID, Caspase-8, NFκB, STAT3, JAK1, COX-2, LAMP2, and MAP LC3II. Nanoindentation and atomic force microscopy (AFM) were used to measure dentin hardness and modulus of elasticity. Results: BID expression increased with age, whereas Caspase-8 showed a relative decrease (*p* < 0.05). Anterior teeth exhibited higher marker positivity than molars for BID (*p* = 0.029), Caspase-8 (*p* = 0.004), STAT3 (*p* = 0.001), and JAK1 (*p* = 0.026). Mechanical analysis revealed the lowest modulus of elasticity in young root dentin and the highest in old coronal dentin, reflecting progressive age-dependent structural changes. Conclusions: Aging of the dentin–pulp complex involves coordinated modulation of apoptotic, autophagic, and inflammatory pathways, closely linked to altered mechanical properties. This interplay likely influences pulp vitality and adaptive cellular responses, highlighting potential targets for age-adapted dental therapeutic strategies.

## 1. Introduction

Dental pulp is a specialized connective tissue that maintains tooth vitality [1] and contains a rich vascular and neural network as well as dental pulp stem cells (DPSCs) with regenerative potential [2]. Aging of the dental pulp is a complex, multidimensional process that affects both cellular and extracellular components of the pulp–dentin complex including reduced vascularization, decreased cellularity, fibrosis, and calcification, ultimately limiting regenerative capacity [3]. Contrary to a simple degenerative perspective, age-related changes represent coordinated adaptations involving autophagy, apoptosis, inflammation, and vascular remodeling [4]. These processes collectively regulate pulp vitality, regenerative potential, and tissue homeostasis.

Recent studies highlight the importance of NFκB, JAK/STAT, and mitochondrial apoptotic pathways in pulp regulation, while autophagy markers such as MAP LC3-II and LAMP2 ensure maintenance of cellular homeostasis [5,6] and their expression has been linked to cellular adaptation to oxidative and inflammatory stress [7]. On the other hand, BID, a pro-apoptotic member of the Bcl-2 family, plays a central integrative role in the apoptotic signaling pathway. Following proteolytic activation by caspase-8 [8], it carries signals from the external pathway to the mitochondrial (internal) pathway of apoptosis [9]. In addition to its role in apoptosis, BID also functions as a key “node” in the network of cell death, as it participates in the cross-regulation between apoptosis, necroptosis, and pyroptosis, highlighting its importance as an integrative pro-apoptotic mediator [10].

While physiological levels of apoptosis ensure the elimination of damaged cells and maintenance of pulp integrity, overactivation accelerates degeneration and limitation of regenerative capacity [11]. Age-related changes in pulp are often associated with increased apoptosis and decreased autophagic activity, leading to an imbalance between cell survival and cell death [12]. Inflammation is a key regulator that integrates autophagy and apoptosis [13]. COX-2 and NF-κB regulate the expression of pro-inflammatory cytokines, such as IL-1β, TNF-α, and IL-6 [14,15,16], while the JAK/STAT3 signaling pathway influences both stem cell survival [17] and chronic inflammatory responses [18]. Persistent activation of these pathways may shift the balance from tissue maintenance toward degeneration. In parallel, CD34-positive progenitor cells are critical for vascular support and regenerative potential [19], yet their functionality appears to decline with age.

Despite growing interest in pulp aging, microstructural changes in dentin and mechanical properties in human teeth remain underexplored. Dental hardness and elasticity are closely related to functional integrity and longevity of teeth, maintaining masticatory function and quality of life. Modern high-resolution techniques provide a unique opportunity for in-depth study of structural and functional changes in pulp tissue. Nanoindentation and Atomic Force Microscopy (AFM) provide nanometric resolutions to evaluate cell-surface interactions and ultrastructural changes [20]. Nanoindentation provides a quantitative assessment of the mechanical properties of pulp tissue, which reflect the degree of aging, remodeling, and loss of vitality [21]. Mechanically, dentin undergoes progressive mineralization, leading to increased hardness and modulus of elasticity in the crown, while root dentin remains relatively more deformable in younger individuals. The age-dependent nanomechanical changes influence stress distribution and may affect odontoblast survival, apoptosis, and autophagic activity. Understanding the interplay between cellular mechanisms and mechanical properties is therefore critical for elucidating the biology of aging in the dentin–pulp complex, reflecting both age-related mineralization and adaptive remodeling.

Importantly, the present study is specifically designed to investigate the association between age and the expression profile of apoptotic, inflammatory, and autophagy-related biomarkers in the dental pulp in the context of preserving pulp vitality and its regenerative potential. A growing number of studies highlight that autophagy and apoptosis are not isolated processes, but closely interrelated pathways that share common regulatory mechanisms [22,23]. Special attention is given to MAP LC3-II as a marker of sustained autophagic activity, allowing us to assess whether autophagy remains functionally active across different stages of aging. Decreased expression of LAMP2 and MAPLC3-II has been reported to be associated with loss of pulp vitality in adult individuals. At the same time, activation of Caspases and BID is associated with increased apoptotic pulp cell death in aging and inflammation [24]. Moreover, emphasis is placed on the JAK1/STAT3 signaling axis as a mediator of adaptive remodeling and potential sex-dependent tissue responses. STAT3 and JAK signal proliferative and immunomodulatory changes that may have an impact on pulp stem cells, while the excessive activity of COX-2 and NFκB correlates with chronic inflammatory status [25,26,27].

Importantly, aging is associated with a progressive decline in the functional capacity of CD34+ cells, potentially limiting regenerative potential [28]. This study was designed to address this gap by investigating coordinated age-related alterations in autophagy, apoptosis, inflammation, vascularization, and the mechanical properties of the dentin–pulp complex across different age groups. We hypothesized that these processes are interrelated and jointly contribute to the maintenance of pulp homeostasis during aging.

Accordingly, we analyzed the expression of CD34, Caspase-8, BID, COX-2, LAMP2, MAPLC3-II, NF-κB, STAT3, and JAK in aging dental pulp. By integrating immunohistochemical, nanomechanical, and morphological approaches, we aimed to provide a comprehensive and multilevel characterization of age-associated changes in pulp structure, function, and dentin nanoarchitecture. This integrative framework was intended to clarify the relationship between molecular signaling pathways and the mechanical microenvironment, with potential implications for the preservation of pulp vitality and the development of age-tailored therapeutic strategies in restorative dentistry.

## 2. Materials and Methods

### 2.1. Study Design and Sample Collection

The investigation was designed as a forward-looking observational study and was based on a collection of 90 human teeth obtained during routine dental care. Teeth were obtained from patients undergoing routine dental extractions at the Department of Oral Surgery, Medical University of Plovdiv, performed according to standard clinical care protocols, including periodontal therapy (mainly incisors and premolars), orthodontic treatment (predominantly unerupted or developing third molars), and eruption-related complications involving wisdom teeth. Only morphologically preserved teeth were included. Among the 90 teeth included, 30 were completely unerupted third molars. The overall cohort consisted of 45 male and 45 female patients, evenly distributed across the age groups.

For analytical purposes, the samples were stratified according to the biological stage of the pulp–dentin complex, which correlates with patient age. Three equally sized cohorts were created:

Group 1 (Developing pulp and dentin): Teeth from individuals aged up to 17 years (n = 30);

Group 2 (Mature pulp and dentin): Teeth from individuals aged 18 to 50 years (n = 30);

Group 3 (Aged pulp and dentin): Teeth from individuals older than 51 years (n = 30).

### 2.2. Inclusion and Exclusion Criteria

#### 2.2.1. Inclusion Criteria

Teeth were accepted into the study when they met all of the following conditions:

- Removal was part of standard dental treatment;
- No visible structural damage was present;
- Extraction was indicated by periodontal disease, orthodontic planning, or disturbed tooth eruption;
- The donor belonged to one of the predefined age categories (<18, 18–50, or >51 years).

#### 2.2.2. Exclusion Criteria

Specimens were not used if any of the following were identified:

- Signs of dental decay, previous restorations, or mechanical damage;
- History of endodontic procedures;
- Presence of local pathological processes, including cystic or tumorous lesions;
- Extraction performed due to trauma or for non-medical reasons.

### 2.3. Immunohistochemistry

Paraffin-embedded sections of human pulp–dentin complexes were analyzed by immunohistochemistry. After extraction, following fixation in 10% neutral formalin for 24 h, samples were processed within 24–48 h for decalcification and embedding. To facilitate penetration of the decalcifying agent while preserving protein structures, the enamel and superficial dentin were mechanically removed. The specimens were then decalcified to allow optimal antigen exposure for immunolabeling.

Several decalcification protocols described for bone tissue were initially tested on human dentin, and antigen preservation was evaluated [20]. The protocol providing the best epitope retention was selected for the study. According to this protocol, samples were treated with 3% hydrochloric acid for 2 h, rinsed three times in distilled water for 15 min, and subjected to a second 2 h decalcification in 3% hydrochloric acid. Afterward, tissues were dehydrated, cleared, and embedded in paraffin.

Sections (2.0–2.5 μm) were deparaffinized, rehydrated, subjected to antigen retrieval, and incubated for 1 h at room temperature with monoclonal antibodies—CD34 (B-6, sc-74499), BID (B-3, sc-373939), Caspase-8 (8CSP03, sc-56070), NFκB p65 (F-6, sc-8008), Stat3 (F-2, sc-8019), JAK1 (A-9, sc-1677), COX2 (D-5, sc-514489), LAMP2 (H4B4, sc-188822), and MAP LC3β (G-2, sc-271625)—diluted 1:200 (Santa Cruz Biotechnology Inc., Dallas, TX, USA), using an automated Leica Bond-Max system (Leica Biosystems Nussloch GmbH, Nußloch, Germany) according to the manufacturer’s protocol.

Immunodetection was performed using the Bond Polymer Refine Detection Kit (Leica Biosystems Nussloch GmbH, Nubloch, Germany), based on a horseradish peroxidase (HRP)-linked polymer system with 3,3′-diaminobenzidine (DAB) as chromogen. Sections were counterstained with hematoxylin, dehydrated, and mounted. Negative controls were processed by omission of the monoclonal antibody.

The overall process, including fixation, decalcification, paraffin embedding, and complete IHC analysis of all 90 dentin–pulp samples, took 12 months strictly following the protocols for time exposure of different reagents and more than a year of microscopic examination of different regions of interest, photocopying, grouping and comprehensive comparative evaluation.

Immunostained sections were examined using a Leica DMI3000B light microscope with digital image capture (DFC295; Leica Microsystems Nussloch GmbH, Nußloch, Germany).

### 2.4. Immunohistochemical Evaluation Criteria and Region Selection

For immunohistochemical evaluation, predefined regions of interest (ROIs) were systematically selected in each specimen. These included: (i) the odontoblastic layer, (ii) the subodontoblastic zone, and (iii) the central pulp region. For each case, five non-overlapping high-power fields (400× magnification) were analyzed per region.

The semi-quantitative score was based on both staining intensity and the percentage of positive cells, as follows:

0—no detectable staining in target cells;

1—weak cytoplasmic and/or nuclear staining in <50% of cells within the evaluated field;

2—strong and clearly distinguishable staining in ≥50% of cells.

Only morphologically identifiable pulp cells (odontoblasts, fibroblast-like cells, and endothelial-associated cells where applicable) were evaluated. Background or non-specific staining was excluded from scoring. Representative images were selected from standardized acquisition settings using identical exposure parameters for all samples to ensure comparability. Two blinded observers evaluated all slides independently. Agreement was tested using Cohen’s kappa (κ = 0.79–0.92), and discrepant results were resolved by joint review.

### 2.5. Nanoidentation and Atomic Force Microscope (AFM)

After extraction, the teeth were cleaned using ultrasonic treatment. Samples were then grouped according to donor age and tooth topography (crown/root).

Nanoindentation testing was performed with a universal nanomechanical testing system (Briker Nano GmbH, Berlin, Germany) equipped with a Berkovich-type diamond indenter (three-sided pyramid, tip radius ~100 nm). A total of 48 indentations were made per sample (8640 in total), spaced 80 µm apart. Prior to testing, the instrument was calibrated using a fused silica standard. A Poisson’s ratio of 0.3 was assumed for dentin in Young’s modulus calculations.

The loading protocol consisted of approaching the indenter to the specimen surface, applying a maximum load of 100 mN over 15 s, holding at maximum load for 10 s, reducing the load to 10% within 15 s, holding at this reduced load for 15 s, and then fully unloading. During testing, load–displacement curves were recorded and used to calculate nanohardness and Young’s modulus according to the Oliver–Pharr method [27,28].

The morphology and dimensions of the resulting indentations were further examined using an atomic force microscope (AFM), providing high-resolution three-dimensional imaging for validation of the mechanical data.

### 2.6. Ethics Statement

The study conforms to the Declaration of Helsinki and was approved by the Scientific Ethics Committee at the Medical University of Plovdiv with Protocol number 1/13.02.2020. Extracted teeth are classified as pathological medical waste under national regulations and are not considered organ donation material. Written informed consent was obtained from all participants prior to inclusion in the study.

### 2.7. Statistical Analysis

Statistical analysis was performed to evaluate quantitative variability. For immunohistochemical semi-quantitative data, non-parametric tests were primarily applied due to ordinal scale characteristics. Adjustment for multiple comparisons was performed using the Bonferroni correction. Data distribution was tested for normality (Kolmogorov–Smirnov test) and for homogeneity of variance. Group differences were examined using ANOVA followed by Tukey’s HSD post hoc test, while result validation was carried out with the Dunnett’s T3 test. For non-parametric comparisons among more than two independent groups, the Kruskal–Wallis’s test was applied, and pairwise differences were assessed using the Mann–Whitney U test. These procedures were used for both dependent and independent variables with multiple categories, including regional expression profiles and age-based stratification. Statistical analysis was conducted using IBM SPSS Statistics 30.0 (IBM Corp., Armonk, NY, USA). A *p*-value below 0.05 was considered statistically significant.

## 3. Results

### 3.1. Light Microscopy and CD34 Immunohistochemistry

**Figure 1:**
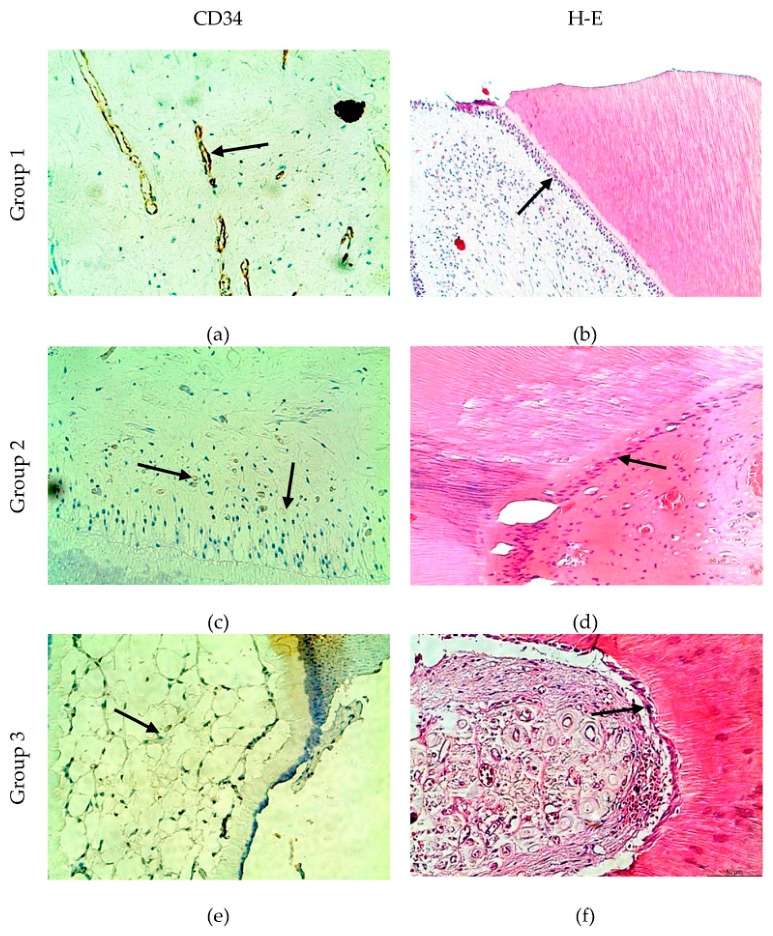
Histological observation by H-E staining and IHC for CD34. (**a**) Rich vascular network with well-formed walls and CD34-positive endothelial cells throughout the pulp (black arrow); (**b**) a palisade of odontoblasts with mineralised methadentin and intensely coloured H-E nuclei (black arrow); (**c**) small blood vessels in the subodontoblastic layer, weaker CD34 expression (black arrow); (**d**) diffuse calcification and obliterated root canal with odontoblasts included, H-E staining (black arrow); (**e**) multi-row palisade and dense vascular network—a sign of angioadaptation, with weak CD34-positivity (black arrow); (**f**) fibrous pulp with single odontoblasts, capillaries and nerve fibers, H-E staining (black arrow); ×200.

The comparative histological and imunohistochemical data are presented in Figure 1.

Light microscopic assessment suggested age-related structural changes within the dental pulp. In young teeth, the pulp generally showed preserved cellularity, multi-layered odontoblasts, continuous predentin, and a relatively rich CD34-positive capillary network (Figure 1a,b). In mature teeth, features consistent with early remodeling were observed, including single-row odontoblasts, fibrous areas, denticles, and calcifications, while vascularization appeared largely preserved (Figure 1c,d).

In older teeth, the pulp more frequently demonstrated pronounced fibrosis, isolated odontoblasts, calcifications, and weaker CD34-positive vascular profiles, particularly in individuals over 70 years of age (Figure 1e,f). CD34-positive microvessels were mainly localized beneath the subodontoblastic zone, with additional distribution throughout the central pulp tissue. Within Group 3 (n = 30), 14 patients were aged 50–60 years, 10 were 61–70 years, and 6 were older than 70 years. Based on these observations, we hypothesized that attenuation of CD34 expression may become more evident with advanced age.

### 3.2. Apoptotic Markers: BID and CASPASE-8

**Figure 2:**
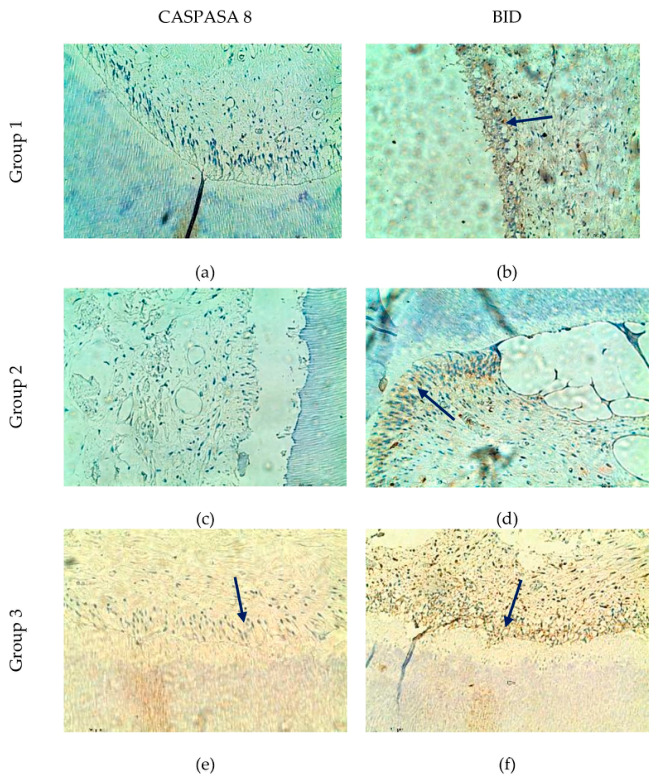
Expression of BID and Caspase-8 in dental pulp odontoblasts. (**a**) A negative immunohistochemical reaction to Caspase-8. (**b**) Immunopositive marking for BID. (**c**) Negative immunohistochemical reaction to Caspase-8. (**d**) Immunopositive marking for BID in the pulp horn. (**e**,**f**) Immunopositive marking for BID and Caspase-8; immunopositive marking (blue arrows); ×200.

Our results demonstrated age-related differences in BID and Caspase-8 expression patterns. BID immunoreactivity appeared more frequent and widespread in odontoblasts and pulpal cells, whereas Caspase-8 expression was more variable and predominantly localized in the coronal pulp region (Figure 2). These observations may suggest a greater involvement of mitochondrial apoptotic signaling in aging pulp tissue, while caspase-8-mediated pathways may be activated under more specific cellular stress conditions.

The distribution of BID and Caspase-8 expression according to demographic and anatomical variables is presented in Table 1.

After analysis of BID and Caspase-8 expression from Table 1, no statistically significant associations were observed with age or sex (*p* > 0.05). With respect to tooth type, higher immunopositivity was noted in anterior teeth for both BID (*p* = 0.029) and Caspase-8 (*p* = 0.004). No significant differences were identified between maxillary and mandibular teeth. According to anatomical region, there was a tendency toward higher expression in the crown compared with the root. For BID, this trend did not reach statistical significance, whereas Caspase-8 showed significantly higher expression in the crown region (*p* = 0.042).

Overall, these findings suggest that BID and Caspase-8 expression may be more closely related to tooth type and the anatomical area examined than to age, sex, or jaw location. The relatively higher immunopositivity in anterior teeth may reflect differences in functional loading or local metabolic activity, while increased Caspase-8 expression in the coronal pulp may indicate a greater responsiveness of this region to external stimuli (Table 2).

### 3.3. NFκB, STAT3, and JAK1 Signaling

**Figure 3:**
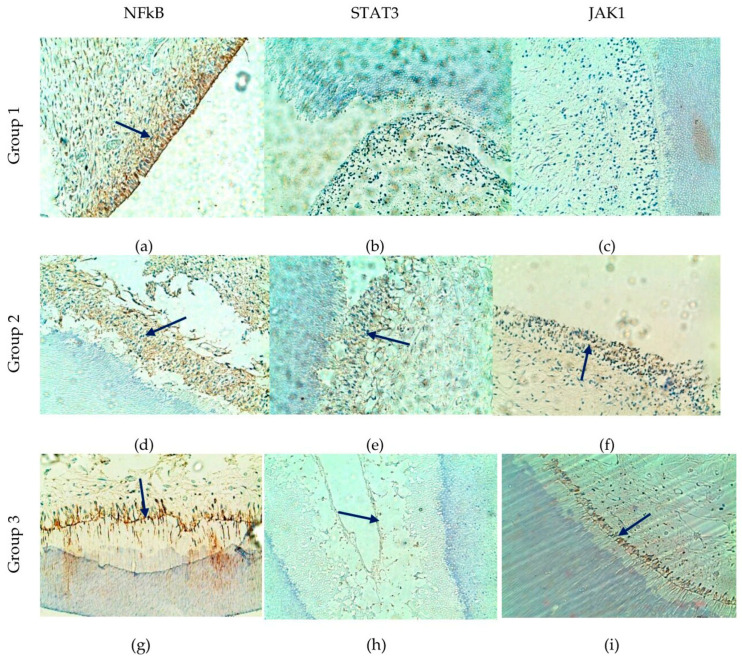
Comparative distribution in the pulp and odontoblasts of antibodies for NFκB, JAK1 and STAT3. (**a**) Immunopositive marking for NFκB in the area of the pulp horn of a tooth embryo. (**b**,**c**) Immunomarkers for JAK1 and STAT3 are negative. (**d**,**e**) Immunopositive marking for NFκB and STAT3; (**f**) weak-positive immune response to JAK1; (**g**) the expression of NFκB is strongly expressed in the odontoblastic and subodontoblastic zones, engages the cytosol and cell membrane; (**h**) the immune response to STAT3 is discrete. (**i**) Immunomarking for JAK1 is positive; immunopositive marking (blue arrows); ×200.

NFκB showed relatively high and widespread expression across different age groups and dental regions, particularly within odontoblastic and subodontoblastic areas (Figure 3a,d,g). STAT3 expression appeared more variable, with a tendency toward increased positivity in older specimens, especially in crown regions and anterior teeth. JAK1 demonstrated a partially similar distribution pattern, although with generally weaker expression levels (Figure 3).

Taken together, these findings may indicate differential activation patterns of NFκB, STAT3, and JAK1 signaling pathways in dental pulp tissue. NFκB expression appeared more consistently distributed, whereas STAT3 and JAK1 immunoreactivity showed greater variability among age groups and anatomical regions.

Table 2 presents the comparative immunoexpression of NFκB, JAK1, and STAT3 in odontoblasts and pulp tissue according to demographic and dental variables.

NFκB demonstrated consistently high immunopositivity across all age groups. In the young pulp, staining was uniformly strong (2+), with no immunonegative cells identified. In the mature pulp, a small proportion of immunonegative odontoblasts (0–7.9%) was observed, whereas most cells (86.8%) remained strongly positive. In the old pulp, NFκB again showed uniformly high positivity (100% score 2+). No statistically significant differences were detected according to sex, tooth type, or jaw position. Topographically, expression appeared comparable between crown and root regions, with a slightly higher proportion of strong positivity in the crown. These findings may suggest that NFκB represents a relatively stable marker in odontoblasts, while the modest reduction observed in mature pulp could reflect a transitional stage of cellular activity.

STAT3 appeared to show age-related variation. In the young pulp, 66.7% of cells were immunonegative, and no cases of strong expression (2+) were observed. In the mature pulp, 26.3% of cells demonstrated strong positivity, while the proportion of negative cells decreased to 44.7%. In the old pulp, 58.3% of samples showed strong positivity. Higher expression was also observed in females (48.8% score 2+) compared with males (25.0%), in anterior teeth (68.4%) compared with molars (28.1%), and in crown pulp (42.1%) compared with root pulp (26.9%). These observations may indicate that STAT3 becomes more evident with age and could be associated with regulatory processes related to pulp adaptation and cellular aging.

JAK1 also showed variation across age groups. In the young pulp, 66.7% of cells were negative, with no cases of strong expression (2+). In the mature pulp, 18.4% showed strong positivity, 31.6% moderate positivity, and the proportion of negative cells decreased to 50.0%. In the old pulp, 36.1% of cells were strongly positive, 44.4% moderately positive, and 19.4% remained negative. Females demonstrated a higher proportion of strong expression than males (34.9% vs. 12.5%), and anterior teeth showed greater positivity than molars (42.1% vs. 18.8%). Differences between crown and root regions did not reach statistical significance. These findings may suggest a gradual increase in JAK1 involvement with age, with a possible role in later adaptive changes of the pulp (Table 2).

### 3.4. Autophagic Markers: MAP LC3-II, LAMP2, and COX2

**Figure 4:**
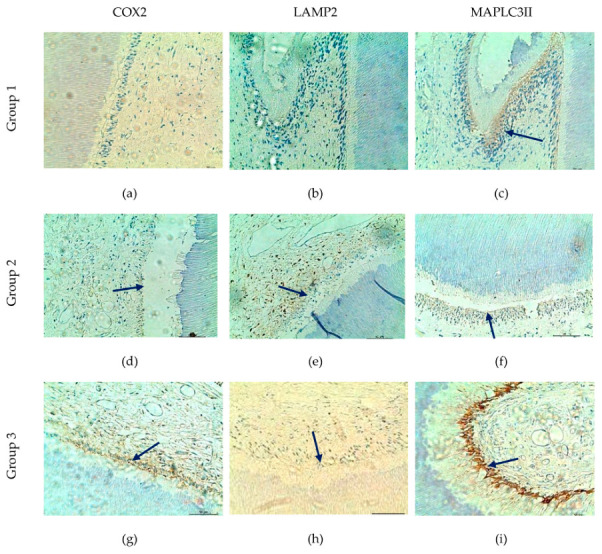
Expression of MAP LC3II, COX2 and LAMP2 in dental pulp. (**a**,**b**) Immunonegative reaction to COX2 and LAMP2. (**c**) Immunopositive marking for MAP LC3II only. (**d**–**f**) Immunopositive co-expression of the three markers (MAP LC3II, COX2 and LAMP2). (**g**) Immunopositive response to COX2. (**h**) Lack of expression for LAMP2. (**i**) Strong positive coloration for MAP LC3II; immunohistochemical staining is indicated by blue arrows; NS (not significant); *p* < 0.05; ×200.

MAP LC3-II showed relatively strong expression across all age groups, which may be consistent with sustained autophagic activity. LAMP2 expression appeared to vary with age, possibly indicating differences in lysosomal fusion processes and autophagic completion, while COX-2 was observed more intermittently, which may reflect localized cellular stress or inflammatory activation (Figure 4).

Table 3 presents the comparative immunoexpression of COX-2, LAMP2, and LC3II in odontoblasts and pulp tissue across demographic and dental variables. Overall, the maintained expression of autophagy-associated markers may suggest a potential contribution of these pathways to the relative resilience of odontoblasts and preservation of pulp vitality despite age-related structural changes.

Analysis of the expression profiles of COX2, LAMP2 and MAP LC3II revealed distinct distribution patterns that appear to vary with age and anatomical location, although no strong statistical associations were identified.

COX2 showed generally low to moderate expression levels, with a gradual increase in the proportion of positive cases across age groups (from 11.1% in younger individuals to 38.9% in older individuals); however this trend did not reach statistical significance (*p* > 0.05). No meaningful differences were observed with respect to gender, tooth type, or anatomical region. These findings may suggest episodic activation of COX2, potentially reflecting age-related changes in mitochondrial quality control processes, although without indicating it as a dominant mechanism.

LAMP2 was expressed more frequently compared to COX2, with an apparent increase in positivity from 33.3% in the young group to 58.3% in the older group, again without statistical significance. The relatively higher expression observed in anterior teeth (68.4% compared to 40.6% in posterior teeth), as well as variability across different pulpal regions, may indicate some degree of regional specificity in autophagolysosomal activity. Overall, these observations are consistent with the notion that LAMP2 may function as an intermediate marker, present under conditions of ongoing autophagic activity, while potentially reduced in states of diminished lysosomal function.

In contrast, MAP LC3II demonstrated the highest and most consistent expression, with positivity observed in the majority of cases (73.7–82.8%) regardless of age, sex, tooth type, jaw, or anatomical location (*p* > 0.05). These results may support the interpretation that MAP LC3II represents a relatively stable marker of autophagic activity, potentially reflecting a continuously active role in maintaining cellular homeostasis and supporting odontoblast survival across different age groups.

In summary, although no statistically strong dependencies were identified, the observed patterns may still suggest subtle age-related and topography-associated differences in organelle turnover and cellular adaptive processes.

### 3.5. Integrated Age-Dependent Marker Expression

**Figure 5:**
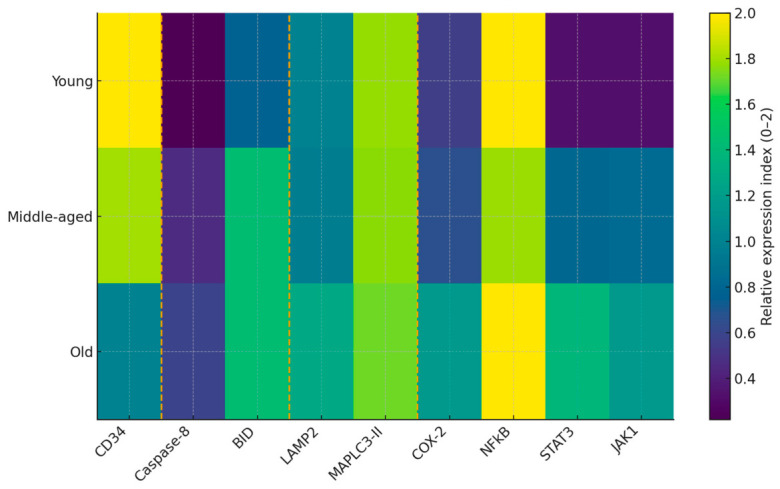
The pooled heatmap visualizes the comparative expression of nine key markers (NFκB, STAT3, JAK1, Caspase 8, COX-2, MAPLC3-II, LAMP2, BID and CD34) in pulp tissue in three age groups (young, mature and old). The color scale (Low–Med–High) shows the degree of immunoexpression.

A pooled heatmap of nine key markers (NFκB, STAT3, JAK1, Caspase-8, BID, MAP LC3-II, LAMP2, COX2, CD34) revealed that NFκB and MAP LC3-II maintain baseline expression, while apoptotic and JAK/STAT pathways are activated in an age-dependent manner. Interestingly, autophagic dynamics (MAP LC3-II, LAMP2) maintain cellular survival, even in older pulp. These data underscore the coordinated regulation of survival, death, and adaptive signaling in the aging pulp (Figure 5).

### 3.6. Nanoindentation and Atomic Force Microscopy (AFM)

Nanoindentation and AFM analyses showed progressive increases in coronal dentin hardness and modulus of elasticity with age, while root dentin remained more deformable in younger samples. AFM fingerprints demonstrated “sink-in” profiles consistent with low plasticity and predominantly elastic responses, aligning with structural observations of sclerotic dentin (available as a Supplementary Figure S1, Table 4).

**Figure 6:**
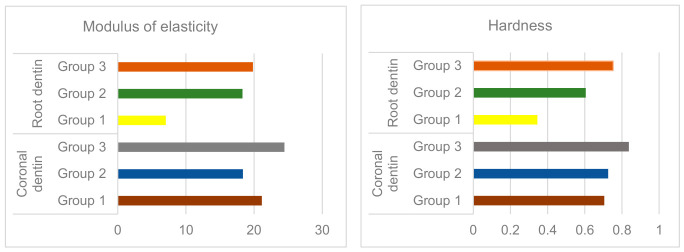
Bar graphs showing modulus of elasticity and hardness in coronal and root dentin across three experimental groups. Note: Statistically significant differences between groups were observed (ANOVA/Kruskal–Wallis test, *p* < 0.001).

Quantitative nanoindentation results are summarized in Table 4 and visualized in Figure 6.

**Figure 7:**
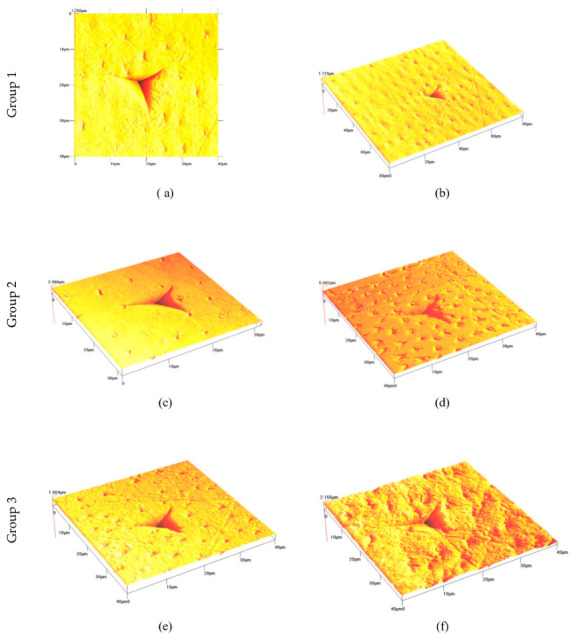
Images obtained by atomic force microscopy: 3D nanoindentation fingerprint on coronary and root respectively for groups (**a**) G1 and (**b**) G4 (young dentin); (**c**) G2 and (**d**) G5 (mature dentin); and (**e**) G3 and (**f**) G6 (old dentin), with a triangular Berkovich indenter. X, Y—horizontal dimensions (μm); Z—depth (μm).

Representative AFM topographic images and nanoindentation fingerprints illustrating the elastic response, plasticity, and fragility of dentin samples across the studied groups are presented in Figure 7.

### 3.7. AFM Images and 3D Nanoindentation Profiles

In addition to calculating the modulus of elasticity and hardness of the samples, atomic force microscope photographs of all groups were also taken. They visualize the nanoindentations and analyze their character, assessing the plasticity and fragility of the samples studied.

Mechanical characterization of dentin reflected age- and region-dependent changes: Coronal dentin showed the highest hardness and modulus of elasticity observed in old specimens (E ≈ 24.4 GPa, H ≈ 0.84 GPa), followed by mature and young groups. Root dentin showed lower stiffness in young pulp (E ≈ 7.05 GPa), increasing with age (Table 4 and Supplementary Figure S1). This age relationship is likely due to the accumulation of transparent dentin in the lumen of the dentin tubules, a process progressing from the apical to the coronary.

In AFM imaging (Figure 7), all groups showed sink-in profiles indicative of low plasticity and predominant elastic response. Harder, more mineralized coronal dentin exhibited the most pronounced elastic behavior, corresponding to increased apoptotic and autophagic signaling.

### 3.8. Summary of Key Findings

Aging of the dentin–pulp complex: key molecular and tissue change.

Aging pulp shows progressive fibrosis, odontoblast loss, calcifications, and vascular variability.

Aging pulp shows progressive fibrosis, odontoblast loss, calcifications, and vascular variability. Intrinsic apoptosis (BID) increases with age, while extrinsic apoptosis (Caspase-8) is intermittent. NFκB remains stable, whereas STAT3 and JAK1 show gradual, age- and region-dependent activation. Autophagy is maintained via MAP LC3-II, while lysosomal function (LAMP2) becomes more variable in older pulp. Dentin mechanical properties increase with age, reflecting coordinated molecular adaptation and coupling between tissue mechanics and cell survival pathways.

## 4. Discussion

Aging of the pulp–dentin complex does not represent passive degeneration but is the result of a complex and multidirectional interaction between autophagy, apoptosis and inflammation. Our data show that it is this molecular balance, unfolded in the context of morphological and mechanical changes in dentin, that determines the vitality and regenerative potential of the pulp. The aging of the pulp–dentin complex is outlined as a multi-layered process in which structural changes in the dentin, adaptive responses to the pulp and the preserved vascular network are intertwined in a complex biological network reflecting the mechanical behavior of the tooth.

In this context, our attention turned to the molecular architecture of pulp vitality, looking at the dynamic interaction between autophagy, apoptosis and vascularization. Autophagy provides cellular cleansing and energy homeostasis by eliminating damaged organelles and proteins, a process that is often compromised with age. Apoptosis, on the contrary, represents a controlled pathway for the elimination of cells, but overactivation can disrupt pulp balance and limit regenerative capacity. Vascularization, in turn, guarantees the supply of oxygen, nutrients and recovery signals, and its reduction is among the earliest signs of pulp aging.

Immunohistochemical analysis of the pulp revealed five molecular response patterns.

### 4.1. NFκB as a Baseline Homeostatic Regulator

NFκB expression was predominantly positive across age groups and pulp regions, which is consistent with a role in maintaining basal homeostatic and immune responsiveness. Although most cells showed strong positivity, slight variations—particularly in the mature group—suggest that its expression may not be entirely uniform throughout life. This pattern generally supports the concept of “inflammatory readiness,” enabling pulp cells to respond to stimuli, while also indicating possible modulation of activity with aging. Our findings are in line with previous reports that associate NFκB activity with both protective responses and, under persistent stimulation, extracellular matrix remodeling and potential tissue changes.

Similar observations of NF-κB activity in pulp tissue have been reported by other authors. Chang et al. (2005) demonstrates that in human pulp stem cells (DPSCs), TNF-α and LPS induce NF-κB activation, highlighting its role in the immune and protective response of the pulp [29]. In a more recent study, Pribadi et al. (2021) found expression of NF-κB in parallel with type I collagen in pulp treated with calcium hydroxide and propolis, suggesting an association between inflammatory status and matrix remodeling [30]. Yu et al. (2022) complement these data by showing that abnormal NF-κB activation in odontoblasts can induce extracellular matrix degradation and accelerate pulp damage [31]. On the other hand, Hozhabri et al. (2015) demonstrate that inhibition of NF-κB in DPSCs exposed to inflammatory cytokines enhances odontoblast differentiation and collagen synthesis, highlighting its dual role—in both protecting and limiting regeneration [32]. Recently, Arora et al. (2022) showed that increased expression of NF-κB was observed in pulpar cells from carious teeth, along with other inflammatory markers, accompanied by changes in the regenerative potential of cells [33].

### 4.2. Age-Dependent Activation of the JAK1–STAT3 Axis

In contrast, JAK1 and STAT3 showed low or absent expression in the young pulp, with a tendency toward increased positivity in the middle-aged and older groups. This increase was more evident for STAT3, particularly in crown regions and anterior teeth, and with higher values observed in females. JAK1 followed a similar but less pronounced pattern, with gradual increases in moderate and strong positivity rather than consistently high expression. These findings suggest a progressive, but not uniform, involvement of the JAK/STAT pathway with aging, possibly reflecting an adaptive response to cumulative cellular stress and tissue remodeling rather than a strictly linear activation process. This interpretation is generally in line with studies in dental and periodontal tissues that report age- and sex-related modulation of STAT3 activity in post-mitotic tissues [34]. Other studies have also associated JAK/STAT signaling, particularly JAK2/STAT3, with bone aging and estrogen deficiency, pointing to potential age- and sex-related differences, although not always consistently across tissues [35,36].

In our cohort, females showed higher STAT3 expression, especially in anterior teeth, which may indicate a tendency toward sex-related differences in pulp response during aging. However, given the sample size and variability in expression levels, this observation should be interpreted with caution. It remains compatible with previous reports suggesting a possible influence of hormonal factors, including estrogen, on JAK/STAT signaling in mineralized tissues, while not allowing definitive conclusions about underlying mechanisms [36].

### 4.3. Apoptotic Pathways: BID and Caspase-8

Apoptotic signaling in our material showed a heterogeneous and moderately age-related pattern. BID expression tended to increase from young to older pulp, with a higher proportion of strongly positive cells in the middle-aged and elderly groups, although without statistically significant differences. This trend may indicate a gradual involvement of mitochondrial-associated apoptotic mechanisms with aging, rather than a strictly progressive or uniform activation. In contrast, Caspase-8 expression remained more variable, with predominantly negative or weakly positive cases in all age groups and only a limited proportion of strongly positive cells, suggesting a less consistent involvement over time.

Topographically, both markers showed higher immunopositivity in anterior teeth, reaching statistical significance, while differences by sex and jaw localization were not significant. For Caspase-8, expression was significantly more frequent in the crown region compared to the root, whereas BID showed a similar tendency without statistical significance. These findings point to a possible influence of local functional and environmental factors, particularly in the crown pulp, rather than a dominant effect of systemic variables such as age or sex.

Taken together, the data suggest that apoptotic activity in odontoblasts does not follow a strictly uniform pattern but rather a context-dependent “mosaic,” in which BID-related mitochondrial signaling becomes more evident with age, while Caspase-8 appears to be engaged more selectively, especially in crown regions exposed to greater external stimuli. This interpretation is broadly consistent with the known role of BID as a mediator linking extrinsic and intrinsic apoptosis pathways [37,38], while also reflecting the variability observed in our results.

### 4.4. Autophagy and Lysosomal Dynamics

Autophagy-related markers (MAP LC3-II and LAMP2) demonstrated an overall preserved activity across all examined age groups, supporting the role of autophagy as a stable mechanism for maintaining intracellular homeostasis in odontoblasts. MAP LC3-II showed consistently high expression in nearly all samples, with only minor variability between demographic or topographic subgroups, suggesting that autophagosome formation remains largely maintained throughout life. In contrast, LAMP2 displayed a more variable pattern, with a tendency toward higher positivity in older samples and in certain regions (e.g., anterior teeth), although without statistically significant differences. This may indicate that while autophagosome formation persists, the efficiency of autophagolysosomal fusion and autophagic completion could be more sensitive to age-related or local microenvironmental factors.

COX-2 expression was generally low to moderate and appeared in an intermittent pattern, with a gradual increase in positive cases with age, although without statistical significance. Its expression did not show consistent dependence on sex, tooth type, jaw, or tooth region. Instead, COX-2 positivity was more localized, suggesting that its activation may reflect episodic mitochondrial or inflammatory stress rather than a continuous basal process [39,40,41].

Overall, these findings suggest a functional separation between a stable autophagy core (LC3-II), a more variable lysosomal component (LAMP2), and a stress-responsive inflammatory marker (COX-2). This pattern may indicate that autophagy remains the dominant homeostatic mechanism in the dental pulp, while inflammatory and mitochondrial stress signals are activated in a more localized and context-dependent manner. Importantly, all examined teeth were collected in the absence of active caries or acute pathology, and some specimens included unerupted third molars with reduced exposure to external stimuli. This likely contributes to the generally low and sporadic COX-2 expression, which may reflect baseline physiological stress rather than overt inflammation.

### 4.5. Vascular Adaptation and Autophagy Sustainability

Despite age-related fibrosis, calcifications, and reduced cellularity, CD34-positive microvessels were still present in most samples, indicating partial preservation of the pulp vascular network. A mild reduction in CD34 expression was mainly observed in individuals over 70 years, but vascular structures remained detectable, especially in deeper pulp areas.

This suggests that, even in advanced age, the pulp maintains a basic level of perfusion that may support residual cellular viability and tissue homeostasis. Such vascular persistence could contribute to sustaining trophic support and limiting the extent of degenerative changes, helping to preserve minimal functional capacity in aged pulp tissue [11,42,43,44].

One of the key observations in this study is that MAP LC3-II remains consistently highly expressed across all examined age groups, without clear dependence on sex, tooth type, jaw, or anatomical region. This pattern, in agreement with previously published data on autophagy as a conserved cellular maintenance mechanism in aging tissues [45,46,47], suggests that autophagosome formation in odontoblasts is stably preserved throughout life. In this context, autophagy appears to represent a continuous homeostatic process supporting cellular integrity in the dental pulp.

At the same time, LAMP2 showed a more variable expression profile, with no consistent statistical pattern across all parameters, but with a tendency toward age-related changes. This may indicate that, while autophagy initiation remains stable, the later stages of autophagic flux, particularly lysosomal fusion and degradation, could be more sensitive to age-related or local mechanical influences. Similar observations have been reported in other studies, where alterations in LAMP2 expression were associated with impaired lysosomal function and reduced efficiency of autophagic degradation [48].

From a broader mechanistic perspective, the current literature highlights the functional interdependence between mitochondria and lysosomes in regulating cellular stress responses and apoptosis in post-mitotic cells [49]. Lysosomal dysfunction, often induced by oxidative or mechanical stress, may contribute to the release of lytic enzymes and subsequent activation of apoptotic pathways, including Bid/Bax-mediated signaling [50,51]. Within this framework, the coexistence of preserved LC3-II expression and variable LAMP2 levels in our samples may help explain why odontoblasts maintain autophagic activity, yet still show signs of age-related vulnerability, such as oxidative stress and lipofuscin accumulation.

Taken together, and in line with previous reports describing LC3-II as a marker of sustained autophagic activity under cellular stress conditions [52,53], our findings suggest that autophagy in the dental pulp is maintained rather than diminished with age. However, the observed variability in LAMP2 expression indicates that the efficiency of autophagic flux may be modulated at the level of lysosomal processing, particularly in older individuals [54].

### 4.6. Nanomechanics in Aged Dentin

In our cohort, nanomechanical analysis revealed that aged coronal dentin exhibited the highest hardness and modulus of elasticity (H ≈ 0.84 GPa; E ≈ 24.4 GPa), whereas young root dentin showed the greatest deformability (E ≈ 7.05 GPa). This pattern reflects a clear age- and region-dependent shift in dentin biomechanics, rather than an isolated or random variation.

Such findings are consistent with previously described characteristics of transparent (sclerotic) dentin, which is generally more mineralized and mechanically harder than normal dentin, but at the same time more brittle and susceptible to crack propagation under mechanical loading [55,56,57]. Published data similarly report higher hardness values in sclerotic dentin (≈0.68 GPa) compared to normal dentin (≈0.57 GPa), alongside increased fragility and altered failure behavior [58,59,60].

Regionally, the observed higher stiffness in coronal dentin compared to root dentin corresponds well with earlier studies, including those of Inoue et al. [61], and may be related to differences in dentinal tubule density and orientation. The lower tubule density in the coronal region has been associated with increased hardness, whereas the higher tubule density in root dentin is generally linked to reduced stiffness and greater compliance [62,63,64,65]. Additional contributing factors likely include variations in the mineral–organic matrix ratio and calcium/phosphorus distribution, which are known to influence dentin mechanical behavior.

The lowest modulus observed in young root dentin may reflect incomplete or ongoing mineralization, while the highest values in aged coronal dentin are in agreement with findings reported by Urabe et al. [66]. It should also be considered that methodological factors, such as applied load, indentation localization (peritubular vs. intertubular dentin), and sample hydration, may contribute to measured variability, as dehydration is known to increase apparent hardness and stiffness [67].

When compared with previously published ranges for elastic modulus in aged coronal dentin, our results show a generally good agreement, particularly regarding increased stiffness in older and more sclerotic regions [68,69]. Minor deviations, such as lower values in young root dentin, can reasonably be attributed to sample-specific factors including third molar mineralization status, local structural heterogeneity, and hydration conditions during testing [68,69].

Mechanical and nanostructural analysis demonstrated clear age- and region-dependent trends in dentin properties. Coronal dentin generally showed higher hardness and modulus of elasticity compared to root dentin, with the most pronounced values observed in older specimens (E ≈ 24.4 GPa; H ≈ 0.84 GPa), while young root dentin exhibited the lowest stiffness (E ≈ 7.05 GPa). These differences indicate a gradual increase in dentin rigidity with age, particularly expressed in the coronal region.

AFM analysis confirmed a predominantly elastic response across all groups, with characteristic “sink-in” indentation profiles consistent with reduced plasticity and increased mineralization (Table 4; Figure S1). This behavior aligns with previously reported age-related changes in dentin, where increased mineral content and sclerosis contribute to higher hardness but also altered mechanical behavior and reduced structural compliance [55,56,57,58,59,60]. Differences between coronal and root dentin are likely related to variations in tubule density and structure, which have been shown to influence mechanical properties in a region-dependent manner [61,62,63,64,65]. Overall, the findings support a gradual structural stiffening of dentin with aging, with clear regional differences between crown and root tissues.

### 4.7. Integrated Molecular–Mechanical Interpretation

Collectively, our results suggest a coordinated, but not strictly linear, adaptation of the pulp–dentin complex during aging.

NFκB appears to maintain a relatively stable baseline activity across samples, which may reflect a constant state of cellular readiness and homeostatic control within dentin-associated tissues. In parallel, the JAK1–STAT3 pathway shows a more variable, age- and region-dependent pattern, which may indicate a gradual involvement in tissue adaptation and remodeling processes over time, rather than uniform activation across all conditions.

BID expression points to a progressive, though heterogeneous, activation of intrinsic apoptotic signaling, particularly in areas potentially exposed to higher functional or mechanical demands. This may represent a controlled response to accumulated cellular stress rather than a generalized apoptotic shift. Autophagy-related markers further support this interpretation: MAP LC3-II remains consistently expressed, suggesting sustained autophagic activity, while the variability of LAMP2 may indicate that the efficiency of autophagic completion is more sensitive to aging-related changes.

Future research should further explore the functional relationships between molecular signaling pathways and the structural–mechanical properties of the pulp–dentin complex using integrated quantitative and correlation-based approaches. In particular, longitudinal and experimentally controlled studies are needed to better clarify causal interactions and to validate the proposed associations observed in the present work, as well as to investigate additional systemic, environmental, and patient-related factors that may influence tissue aging and repair capacity.

This study also has several limitations. The absence of direct statistical correlation analyses between molecular expression and mechanical properties limits the ability to draw definitive mechanistic conclusions. In addition, donor-related systemic conditions were not fully controlled, which may have introduced biological variability. Finally, the use of decalcified specimens and semi-quantitative immunohistochemical assessment, while appropriate for spatial evaluation, restricts precise quantification of protein expression, highlighting the need for future studies employing fully quantitative and multimodal analytical techniques.

## 5. Conclusions

Aging of the pulp–dentin complex is characterized by coordinated molecular and mechanical adaptation rather than simple degeneration. NFκB maintains relatively stable homeostatic activity, while JAK1/STAT3 signaling shows mild age-related activation associated with tissue remodeling. Apoptotic changes are mainly linked to BID-mediated intrinsic pathways, whereas Caspase-8 involvement remains limited and localized. Autophagy is largely preserved through stable MAP LC3-II expression, although variability in LAMP2 suggests reduced lysosomal efficiency with aging. Despite structural aging changes, CD34-positive vasculature persists, supporting residual tissue viability. Mechanically, dentin becomes progressively stiffer and harder with age, especially in the coronal region, reflecting increased mineralization and sclerosis.

Overall, the findings indicate that aging pulp–dentin tissues preserve functional balance through adaptive stress-response and remodeling mechanisms.

## Figures and Tables

**Table 1:** Immunohistochemical profile according to patient and tooth characteristics.

Marker	Factor	Categories	Score 0 (%)	Score 1 (%)	Score 2 (%)	*p*-Value
BID	Age	Young	55.6	11.1	33.3	NS
		Middle-aged	13.9	18.4	57.9	NS
		Old	22.9	19.4	66.7	NS
	Sex	Female	25.6	16.3	58.1	*p* > 0.05
		Male	20.0	20.0	60.0	*p* > 0.05
	Tooth type	Molar	28.1	18.8	53.1	*p* = 0.029
		Anterior	5.3	15.8	78.9	*p* = 0.029
	Jaw	Upper	22.2	15.6	62.2	NS
		Lower	23.7	21.1	55.3	NS
	Tooth part	Crown	22.8	17.5	59.6	*p* > 0.05
		Coronal root	22.2	16.7	61.1	*p* > 0.05
		Apical root	25.0	25.0	50.0	*p* > 0.05
Caspase-8	Age	Young	77.8	22.2	0.0	NS
		Middle-aged	65.8	21.1	13.2	NS
		Old	54.2	32.5	13.3	NS
	Sex	Female	51.2	32.6	16.3	*p* > 0.05
		Male	57.5	32.5	10.0	*p* > 0.05
	Tooth type	Molar	64.1	23.4	12.5	*p* = 0.004
		Anterior	21.1	63.2	15.8	*p* = 0.004
	Jaw	Upper	51.1	37.8	11.1	NS
		Lower	57.9	26.3	15.8	NS
	Tooth part	Crown	45.6	36.8	17.5	*p* = 0.033
		Coronal root	66.7	27.8	5.6	*p* = 0.033
		Apical root	87.5	12.5	0.0	*p* = 0.033

Note: Data are presented as percentages of cases (n) according to immunohistochemical score. Scores: 0 = negative, 1 = weak positivity (<50% of cells), 2 = strong positivity (≥50% of cells). NS (not significant); *p* < 0.05.

**Table 2:** Comparative immunoexpression of NFκB, JAK1, and STAT3 in odontoblasts.

Marker	Factor	Categories	Score 0 (%)	Score 1 (%)	Score 2 (%)	*p*-Value
NFκB	Age	Young	0.0	0.0	100.0	NS
		Middle-aged	7.9	5.3	86.8	NS
		Old	0.0	0.0	100.0	NS
	Sex	Female	0.0	2.3	97.7	*p* > 0.05
		Male	7.5	2.5	90.0	*p* > 0.05
	Tooth type	Molar	4.7	3.1	92.2	*p* > 0.05
		Anterior	0.0	0.0	100.0	*p* > 0.05
	Jaw	Upper	6.7	0.0	93.3	NS
		Lower	0.0	5.3	94.7	NS
	Tooth part	Crown	1.8	1.8	96.5	*p* > 0.05
		Coronal root	7.7	3.8	88.5	*p* > 0.05
STAT3	Age	Young	66.7	33.3	0.0	NS
		Middle-aged	44.7	28.9	26.3	NS
		Old	19.4	22.2	58.3	NS
	Sex	Female	23.3	27.9	48.8	*p* = 0.008
		Male	50.0	25.0	25.0	*p* = 0.008
	Tooth type	Molar	43.8	28.1	28.1	*p* = 0.001
		Anterior	10.5	21.1	68.4	*p* = 0.001
	Jaw	Upper	28.9	24.4	46.7	NS
		Lower	44.7	28.9	26.3	NS
	Tooth part	Crown	28.1	29.8	42.1	*p* = 0.045
		Root	53.8	19.2	26.9	*p* = 0.045
	Tooth part	Crown	28.1	29.8	42.1	*p* = 0.045
		Coronal root	53.8	19.2	26.9	*p* = 0.045
JAK1	Age	Young	66.7	33.3	0.0	NS
		Middle-aged	50.0	31.6	18.4	NS
		Old	19.4	44.4	36.1	NS
	Sex	Female	27.9	37.2	34.9	*p* = 0.011
		Male	50.0	37.5	12.5	*p* = 0.011
	Tooth type	Molar	43.8	37.5	18.8	*p* = 0.026
		Anterior	21.1	36.8	42.1	*p* = 0.026
	Jaw	Upper Lower	42.2	33.3	24.4	NS
			33.2	42.1	23.7	NS
	Tooth part	Crown	33.3	40.4	26.3	*p* > 0.05
		Coronal root	50.0	30.8	19.2	*p* > 0.05

Note: The percentages and *p*-values are indicated in statistical comparisons for antibodies NFκB, JAK1 and STAT3 in the pulp tissue and odontoblastic layer, analyzed by age, sex, tooth type, jaw and part of the tooth. Scores: 0 = negative, 1 = weak positivity (<50% of cells), 2 = strong positivity (≥50% of cells); NS (not significant); *p* < 0.05.

**Table 3:** Immunoexpression of COX2, LAMP2, and LC3II.

Marker	Factor	Categories	Score 0 (%)	Score 1 (%)	Score 2 (%)	*p*-Value
COX2	Age	Young	55.6	33.3	11.1	NS
		Middle-aged	55.3	23.7	21.1	NS
		Old	22.2	38.9	38.9	NS
	Sex	Female	32.6	32.6	34.9	*p* > 0.05
		Male	50.0	30.0	20.0	*p* > 0.05
	Tooth type	Molar	45.3	29.7	25.0	*p* > 0.05
		Anterior	26.3	36.8	36.8	*p* > 0.05
	Jaw	Upper	44.4	35.6	20.0	NS
		Lower	36.8	26.3	36.8	NS
	Tooth part	Crown	44.4	35.6	20.0	NS
		Coronal + Apical root	36.8	26.3	36.8	NS
LAMP2	Age	Young	33.3	33.3	33.3	NS
		Middle-aged	42.1	18.4	39.5	NS
		Old	22.2	19.4	58.3	NS
	Sex	Female	32.6	16.3	51.2	*p* > 0.05
		Male	32.5	25.0	42.5	*p* > 0.05
	Tooth type	Molar	34.4	25.0	40.6	*p* > 0.05
		Anterior	26.3	5.3	68.4	*p* > 0.05
	Jaw	Upper	31.1	20.0	48.9	NS
		Lower	34.2	21.1	44.7	NS
	Tooth part	Crown	31.1	20.0	48.9	NS
		Coronal + Apical root	34.2	21.1	44.7	NS
MAP LC3II	Age	Young	22.2	0.0	77.8	NS
		Middle-aged	5.3	13.2	81.6	NS
		Old	8.3	11.1	80.6	NS
	Sex	Female	4.7	16.3	79.1	*p* > 0.05
		Male	12.5	5.0	82.5	*p* > 0.05
	Tooth type	Molar	9.4	7.8	82.8	*p* > 0.05
		Anterior	5.3	21.1	73.7	*p* > 0.05
	Jaw	Upper	6.7	13.3	80.0	NS
		Lower	10.5	7.9	81.6	NS
	Tooth part	Crown	6.7	13.3	80.0	*p* > 0.05
		Coronal + Apical root	10.5	7.9	81.6	*p* > 0.05

Note: Summary of COX2, LAMP2, and MAP LC3II immunoexpression across age groups, sex, tooth type, jaw, and tooth part. Scores: 0 = negative, 1 = weak positivity (<50% of cells), 2 = strong positivity (≥50% of cells).

**Table 4:** Modulus of Elasticity and Hardness Values for Groups 1–3.

Samples		Modulus of Elasticity				Hardness			
		Mean	Me	SD	*p*-Value	Mean	Me	SD	*p*-Value
Coronal dentin	Group 1	21.135	21.242	1.169	*p* < 0.001	0.705	0.705	0.071	*p* = 0.09
	Group 2	18.364	18.258	0.440	*p* < 0.001	0.726	0.724	0.029	*p* < 0.001
	Group 3	24.446	24.450	0.587	*p* < 0.001	0.837	0.836	0.040	*p* < 0.001
Root dentin	Group 1	7.052	7.044	0.801	*p* < 0.001	0.345	0.354	0.053	*p* < 0.001
	Group 2	18.300	18.233	0.767	*p* = 0.78	0.605	0.598	0.049	*p* < 0.001
	Group 3	19.823	19.744	0.946	*p* < 0.001	0.753	0.746	0.053	*p* < 0.001

Note: The mean values (Mean), median (Me) and standard deviation (SD) of the modulus of elasticity and stiffness measured for each group are presented.

## Data Availability

The data presented in this study are available on request from the corresponding author due to privacy.

## Supplementary Materials

The following supporting information can be downloaded at: https://www.mdpi.com/article/10.3390/life16050844/s1, Supplementary Figure S1: Nanoindentation profiles of dentin across age groups.
