# Supplementary material for: Mechanobiological and Molecular Alterations in the Aging Dentin–Pulp Complex
Source: Life (Basel). 2026 May 20;16(5):844. doi: 10.3390/life16050844 (PMC13208254; doi:10.3390/life16050844)
Supplement: Supplementary file 1 [file life-16-00844-s001.zip › life-4273512-suplementary.pdf]

Figure S1. Nanoindentation profiles of dentin across age groups.

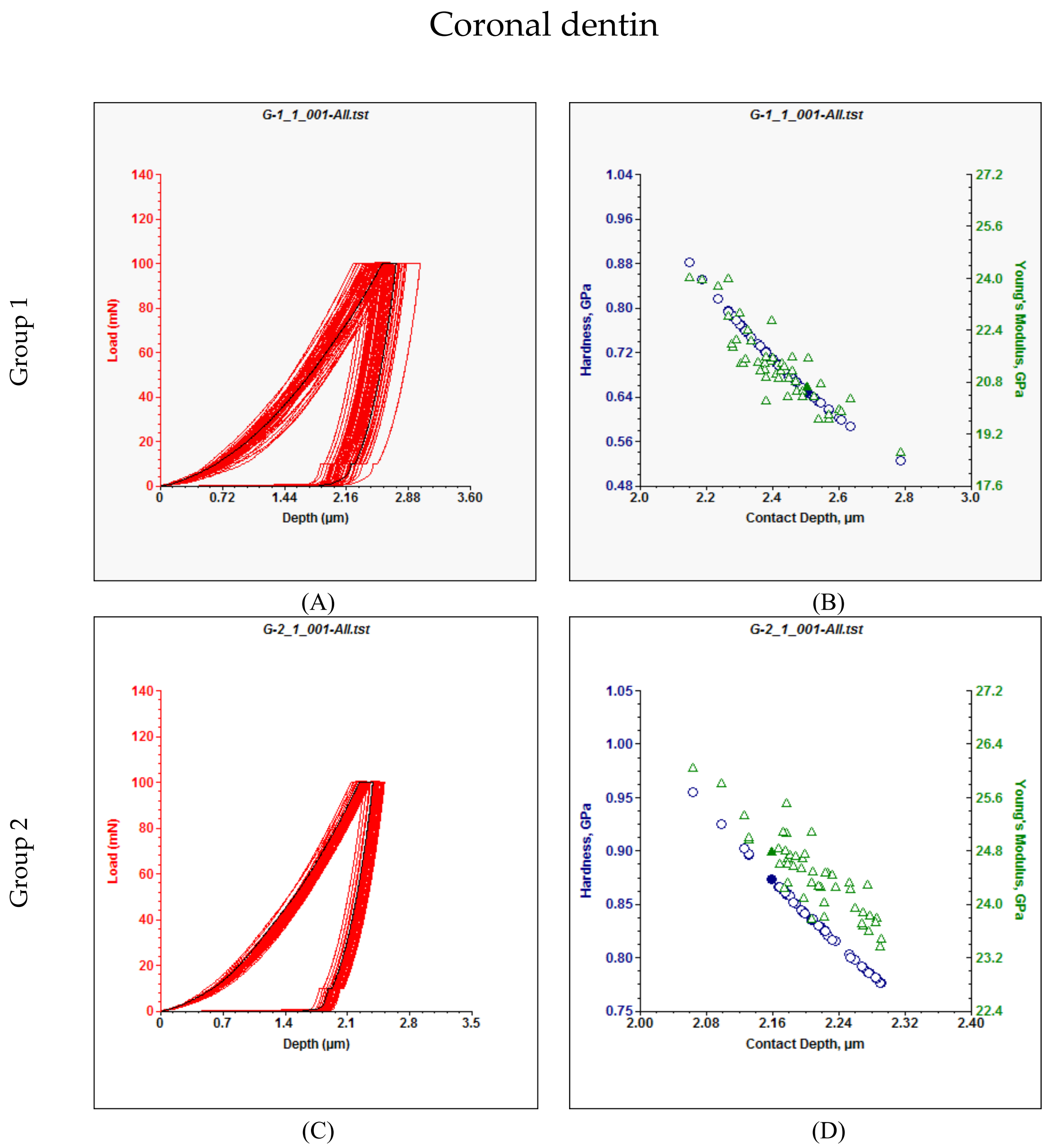

Group 3

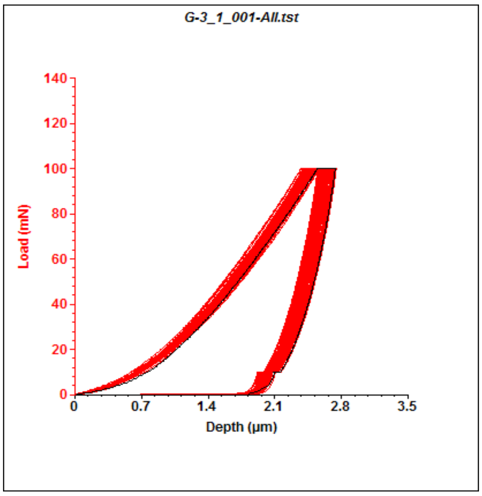

(E)

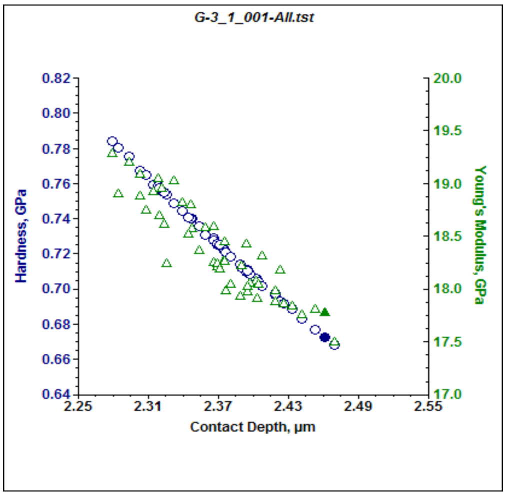

(F)

Root dentin

Group 1

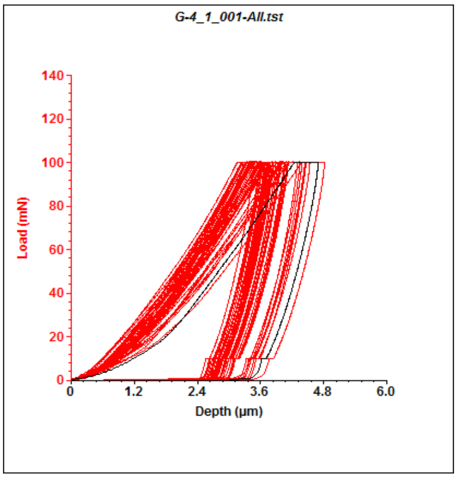

(a)

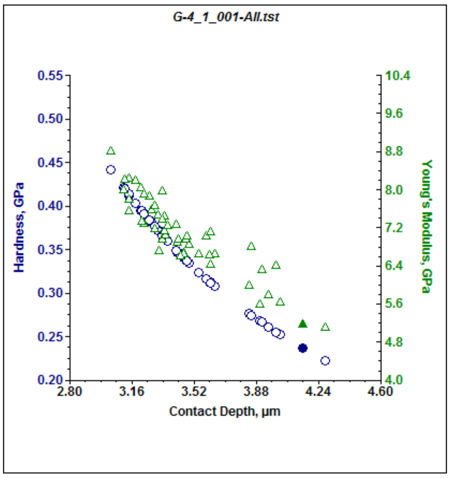

(b)

Group 2

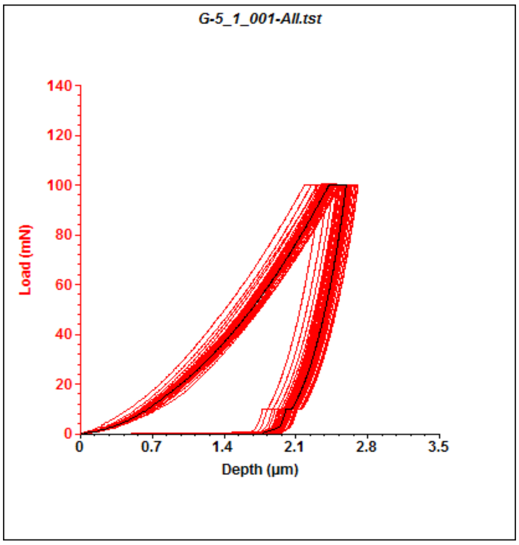

(c)

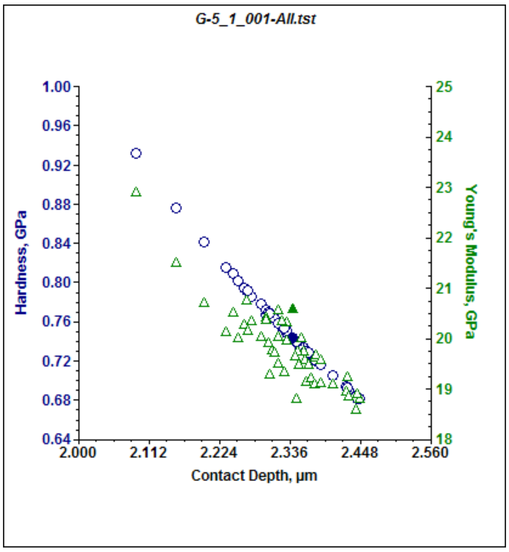

(d)

Group 3

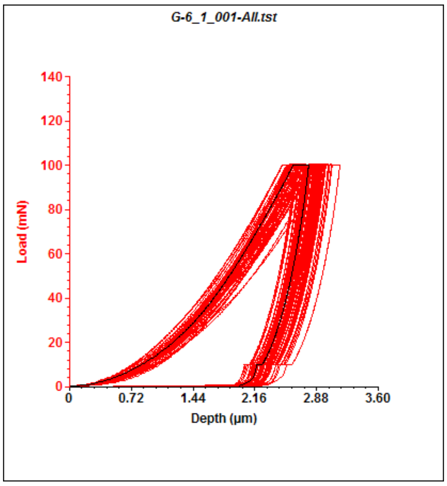

(e)

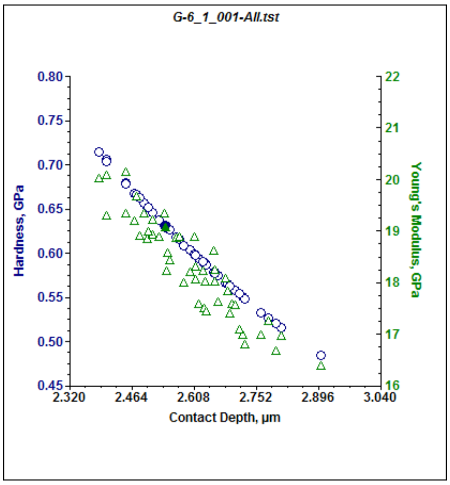

(f)

Note: Coronal dentin : A) Load/depth of penetration curves for G1; (B) values of modulus of elasticity (Δ) and hardness (○) for young coronary dentin; C) Load/depth of penetration curves for G2; (D) values of modulus of elasticity (Δ) and hardness (○) for mature coronary dentin; E) Load/depth of penetration curves for G3; F) values of modulus of elasticity (Δ) and hardness (○) for old coronary dentin.

Root dentin : a) Load curves/depth of penetration for G4; (b) values of modulus of elasticity (Δ) and hardness (○) for young root dentin; c) Load/depth of penetration curves for G5; (d) values of modulus of elasticity (Δ) and hardness (○) for mature root dentin); e) Load/depth of penetration curves for G6; f) values of modulus of elasticity (Δ) and hardness (○) for old root dentin.
